# Marchiafava–Bignami Disease and Pellagra in a Chronic Alcohol User: A Reversible Case of Cognitive Impairment

**DOI:** 10.7759/cureus.102149

**Published:** 2026-01-23

**Authors:** Diogo Ramos, André Valente, Rodrigo Leão, Ana M Serrano

**Affiliations:** 1 Internal Medicine, Unidade Local de Saúde de São José, Lisbon, PRT; 2 Internal Medicine, NOVA Medical School, Lisbon, PRT

**Keywords:** acute reversible encephalopathy, chronic alcohol abuse, marchiafava-bignami disease (mbd), niacin deficiency, pellagra

## Abstract

Marchiafava-Bignami disease (MBD) is a rare complication of chronic alcoholism characterized by demyelination of the corpus callosum. Pellagra, resulting from niacin (vitamin B3) deficiency, typically presents with dermatitis, diarrhea, and dementia. Both MBD and pellagra are often associated with severe nutritional deficiencies. We describe the case of a 56-year-old man with alcohol use disorder admitted after a syncopal episode, presenting with disorientation, apathy, and photosensitive desquamative lesions on sun-exposed skin. Brain MRI revealed T2 hyperintensity in the splenium and body of the corpus callosum, compatible with subacute MBS. Laboratory tests confirmed niacin deficiency, establishing concomitant pellagra. The patient received B-complex vitamin supplementation and was maintained in alcohol abstinence. His cognitive and dermatologic condition improved markedly, with near-complete recovery after six months. This case underlines the importance of recognizing nutritional deficiencies in alcohol-related neuropsychiatric presentations.

## Introduction

Marchiafava-Bignami disease (MBD) is an exceedingly rare alcohol-related demyelinating disorder primarily affecting the corpus callosum, with only approximately 150 cases reported in the literature, and can have a heterogeneous clinical presentation ranging from acute coma to mild cognitive dysfunction [1,2]. Pellagra, caused by niacin deficiency or a disruption of its metabolism, is also rare in industrialized countries but persists in vulnerable populations with prevalence rates of approximately 1% among alcohol-dependent patients, manifesting as dermatitis with pronounced photosensitivity, gastrointestinal symptoms, and neuropsychiatric ailments [3,4]. The coexistence of both conditions is exceedingly rare, with only a single postmortem case documented in the medical literature and no reports in comprehensive reviews of over 150 CT/MRI-confirmed MBD cases or extensive pellagra case series [5]. This case illustrates how dermatologic clues and neuroimaging guided the diagnosis and led to a reversible outcome.

## Case presentation

**Figure 1:**
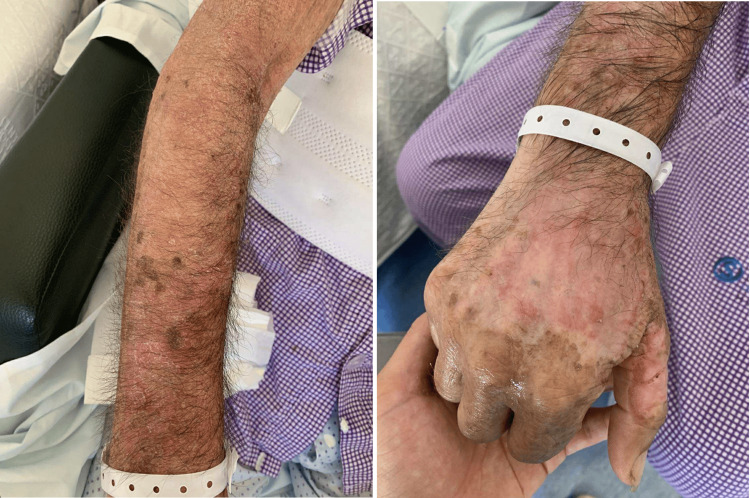
Skin lesions of the upper right arm and hand.

A 56-year-old male construction worker with a long-standing history of alcohol use disorder was admitted following a witnessed syncopal episode without prodromes. Although the exact quantity of alcohol intake could not be reliably determined, daily heavy alcohol consumption (>50 g alcohol/day) over several years was reported by family members. They also described several months of progressive apathy, confusion, and weight loss due to reduced food intake. On examination, he had erythematous, scaly lesions on the dorsal hands and neck in photo-exposed areas (Figure 1).

On neurological examination, the patient was mildly disoriented in time, with impaired attention and reduced processing speed. He exhibited apathy, decreased spontaneous verbal output, and slow, hesitant speech, without clear aphasia. Executive dysfunction was suggested by difficulties in maintaining goal-directed behavior and reduced mental flexibility during the bedside examination. Motor examination revealed intermittent myoclonus of the left upper limb, with no focal weakness. The syncopal episode was not accompanied by features suggestive of a seizure and was clinically considered more consistent with a vasovagal event, possibly related to dehydration and poor nutritional status, rather than a direct manifestation of MBD.

**Figure 2:**
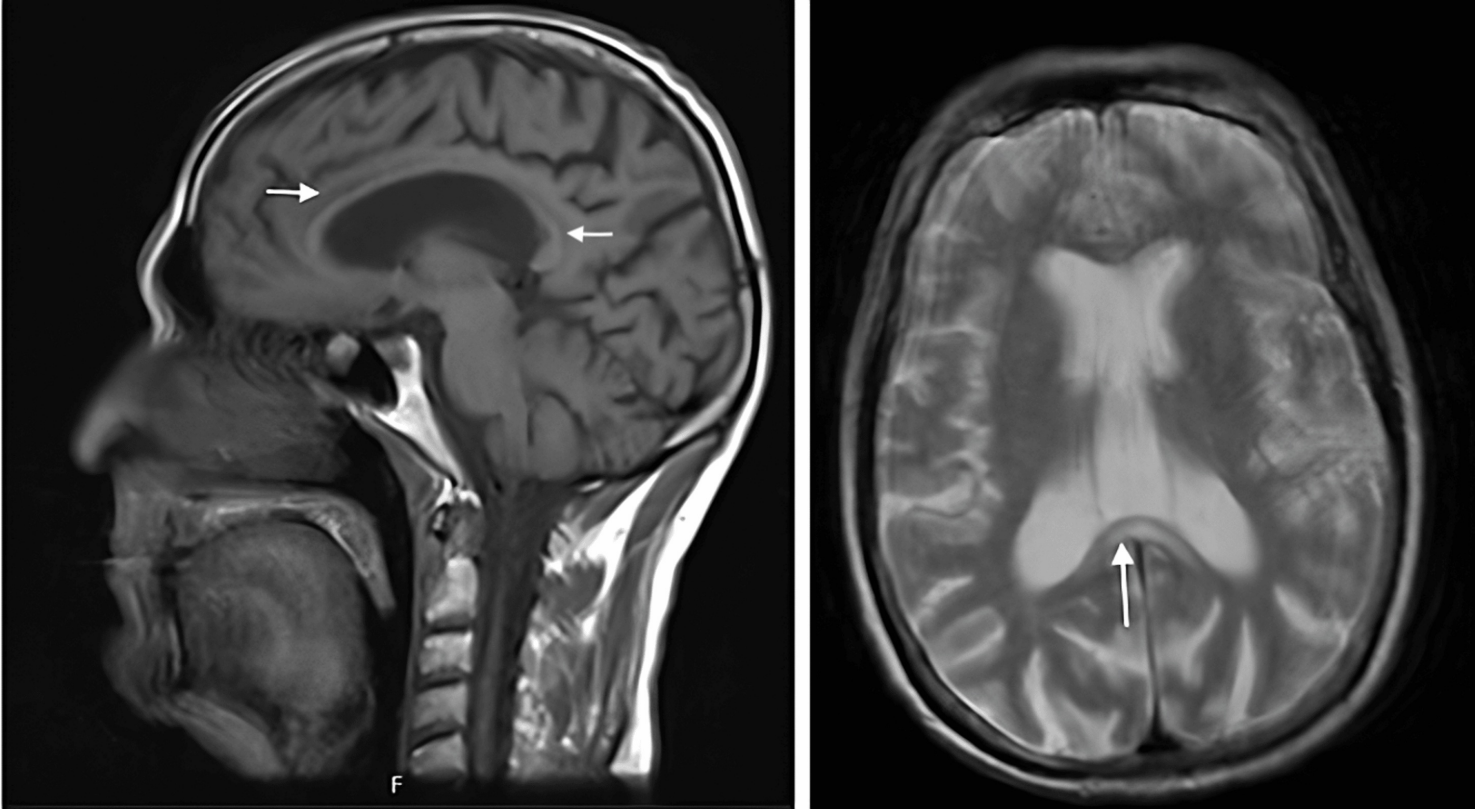
Brain MRI demonstrating marked atrophy of the corpus callosum, particularly involving the body and splenium, with hypointensity on T1 and hyperintensity on T2-weighted images.

Blood analysis revealed macrocytic anemia (hemoglobin, 12 g/dL; mean corpuscular volume 107 fL), thrombocytopenia (122 × 10^9^/L), and elevated liver enzymes (gamma-glutamyl transferase 419 U/L). Serum levels of vitamin B12 (553 pg/mL) and folic acid (3.1 ng/mL) were normal at admission, and vitamin B3 (niacin) was markedly reduced at 9.3 µg/L Nevertheless, given the clinical context of chronic alcohol use and malnutrition, empirical supplementation with B-complex vitamins was promptly initiated. Lumbar puncture was unremarkable (5 leukocytes/mm³; glucose, 80 mg/dL; protein, 27 mg/dL; and lactate dehydrogenase, <30U/L). Electroencephalography showed diffuse slowing without epileptiform activity. Brain MRI demonstrated marked atrophy of the corpus callosum, particularly involving the body and splenium, with hypointensity on T1 and hyperintensity on T2-weighted images, consistent with subacute Marchiafava-Bignami syndrome (Figure 2).

Mini-Mental State Examination (MMSE) score was 13/30, and formal neuropsychological assessment demonstrated severe, diffuse cognitive impairment, with prominent deficits in attention, memory (immediate, working, verbal, visual, and associative), and executive functioning, reflecting impaired encoding, retrieval, planning, cognitive flexibility, and processing speed. Language was marked by anomia with paraphasias and impaired comprehension, despite preserved repetition. Additional impairments included visuoconstructive abilities, calculation, abstraction, and instrumental activities of daily living, accompanied by behavioral changes such as apathy, anxiety, disinhibition, impulsivity, and reduced frustration tolerance.

Dermatological examination revealed erythema extending from the dorsum of both hands circumferentially to the distal third of the forearms, associated with shiny scaling in some areas, as well as cervical erythema. No mucosal involvement was observed. This distribution and morphology were consistent with a photosensitive dermatitis suggestive of pellagra, supported by a markedly reduced niacin serum level.

The patient received intravenous thiamine, pyridoxine, cyanocobalamin, and niacin, followed by oral supplementation on discharge. Alcohol abstinence was maintained, and cognitive assessment one month after discharge still showed significant deficits in attention, memory, and executive function, with apathy and disinhibition.

After six months of treatment and alcohol abstinence, he exhibited substantial improvement (MMSE score was 23/30) and cutaneous lesions had fully resolved. Follow-up neuropsychological assessment revealed mild residual deficits, including slight impairment in attention, verbal memory dysfunction, mild alteration in written calculation, reduced mental control, a mild dysexecutive syndrome, and subtle impairment in instrumental activities of daily living. Overall, a very significant improvement was observed compared with the previous evaluation performed.

## Discussion

This case demonstrates the intersection between chronic alcoholism, nutritional deficiency, and reversible structural brain damage. MBD results from demyelination and necrosis of the corpus callosum, commonly linked to alcohol-related malnutrition and thiamine deficiency [1,2]. Given its heterogeneous clinical presentation, ranging from acute coma to mild cognitive dysfunction, MBD may be misdiagnosed as other neurocognitive disorders, causing delays in diagnosis and treatment that result in poor prognosis [2,5]. This highlights the importance of MRI in patients presenting with behavioral disturbances and neurological findings, as it reveals specific diffusion restriction patterns and symmetric T2/fluid-attenuated inversion recovery hyperintensity in the corpus callosum that establishes the correct diagnosis [6].

Pellagra, resulting from niacin deficiency, may coexist in the same clinical setting as MBD, and the presence of characteristic cutaneous lesions, photosensitive dermatitis with a sharp demarcation, can provide early diagnostic clues in patients with neuropsychiatric symptoms [4,7]. The neuropsychiatric manifestations of pellagra, including dementia, delirium, depression, and encephalopathy, overlap significantly with those of MBD, but pellagra lacks pathognomonic neuroimaging findings; brain imaging is typically unremarkable [6,8]. At an early stage of pellagra, adequate treatment with niacin supplementation results in complete resolution of symptoms, making early nosological diagnosis the most critical stage of management. In contrast, MBD may have a more variable prognosis, with outcomes ranging from complete recovery to persistent neurological deficits depending on the extent of callosal involvement and the timeliness of thiamine and supportive therapy [7-9].

Previous case reports suggest that the simultaneous occurrence of MBS and pellagra is rare but pathophysiologically plausible [5]. Both reflect metabolic brain injury secondary to vitamin depletion and alcohol toxicity. Early recognition and prompt replacement of B vitamins can result in substantial neurological recovery.

## Conclusions

This case highlights a rare but clinically significant coexistence of MBD and pellagra in a patient with chronic alcohol use disorder, emphasizing the profound neurological impact of severe nutritional deficiency. The presence of characteristic photosensitive dermatologic lesions provided an important diagnostic clue, allowing integration of clinical, laboratory, and neuroimaging findings into a unifying diagnosis. Early recognition of these conditions, supported by timely brain MRI and prompt replacement of B-complex vitamins, can result in substantial neurological recovery despite evidence of structural brain involvement. This case underscores the need for a high index of suspicion for reversible nutritional encephalopathies in alcohol-related neuropsychiatric presentations and reinforces the critical role of early diagnosis and intervention.

## References

[1] Koeppen AH, Barron KD (1978). Marchiafava-Bignami disease. Neurology.

[2] Hillbom M, Saloheimo P, Fujioka S, Wszolek ZK, Juvela S, Leone MA (2014). Diagnosis and management of Marchiafava-Bignami disease: a review of CT/MRI confirmed cases. J Neurol Neurosurg Psychiatry.

[3] Li R, Yu K, Wang Q, Wang L, Mao J, Qian J (2016). Pellagra secondary to medication and alcoholism: a case report and review of the literature. Nutr Clin Pract.

[4] Hegyi J, Schwartz RA, Hegyi V (2004). Pellagra: dermatitis, dementia, and diarrhea. Int J Dermatol.

[5] Romero-López J, Moreno-Carretero MJ, Escriche-Jaime D, Corredera-García E, Navarro Fernández-Balbuena C (1997). [The association of Marchiafava-Bignami disease, cerebral pellagra and cerebellar degeneration in an alcoholic patient]. Rev Neurol.

[6] Paidipati Gopalkishna Murthy K (2014). Magnetic resonance imaging in marchiafava-bignami syndrome: a cornerstone in diagnosis and prognosis. Case Rep Radiol.

[7] Fernandes LM, Bezerra FR, Monteiro MC (2017). Thiamine deficiency, oxidative metabolic pathways and ethanol-induced neurotoxicity: how poor nutrition contributes to the alcoholic syndrome, as Marchiafava-Bignami disease. Eur J Clin Nutr.

[8] López M, Olivares JM, Berrios GE (2014). Pellagra encephalopathy in the context of alcoholism: review and case report. Alcohol Alcohol.

[9] Narasimha VL, Ganesh S, Reddy S (2019). Pellagra and alcohol dependence syndrome: findings from a tertiary care addiction treatment centre in India. Alcohol Alcohol.

